# Supplementary material for: ProteinShader: illustrative rendering of macromolecules
Source: BMC Struct Biol. 2009 Mar 30;9:19. doi: 10.1186/1472-6807-9-19 (PMC2672931; doi:10.1186/1472-6807-9-19)
Supplement: Additional file 1 — ProteinShader program without source code. This compressed file contains the complete ProteinShader program including associated libraries, but no source code. A README.txt file gives an overview of the ProteinShader distribution, and the index.html file in the help subdirectory has directions on getting started with the program as well as a set of tutorials. [file 1472-6807-9-19-S1.zip › ProteinShader-beta-0_9_4-binary/help/api/org/proteinshader/structure/io/package-summary.html]

org.proteinshader.structure.io (ProteinShader API)


|  |  |  |
| --- | --- | --- |
| **Overview** \| **Package** \| Class \| **Use** \| **Tree** \| **Deprecated** \| **Index** \| **Help** | |  |
| **PREV PACKAGE**   **NEXT PACKAGE** | **FRAMES**    **NO FRAMES**     **All Classes** |  |


---

## Package org.proteinshader.structure.io

Holds the classes needed for reading Protein Data Bank structure files.

**See:**
  
          **Description**

| **Interface Summary** | |
| --- | --- |
| **StructureReader** | StructureReader provides an interface for reading a protein structure from a file. |

| **Class Summary** | |
| --- | --- |
| **BetaStrandRecord** | This class is used by the PDBLineParser to temporarily store SHEET records. |
| **HelixRecord** | This class is used by the PDBLineParser to temporarily store HELIX records. |
| **PDBAtomFieldExtractor** | Extracts fields from a PDB ATOM or HETATM record. |
| **PDBBetaStrandFieldExtractor** | Extracts fields from a PDB SHEET record. |
| **PDBConnectFieldExtractor** | Extracts fields from a PDB CONECT record. |
| **PDBHelixFieldExtractor** | Extracts fields from a PDB HELIX record. |
| **PDBLineParser** | Parses a line from a PDB file and adds it to a Structure. |
| **PDBStructureReader** | This concrete implementation of interface StructureReader reads a protein structure entry from a '.pdb' file formated according to the PDB Contents Guide (Version 2.2, 20 Dec 1996) and creates a Structure. |
| **TubeVertices** | Creates the vertices for a segment of a three-dimensional tube by using a waist polygon that approximates a circle as the number of vertices in the waist polygon becomes large. |

## Package org.proteinshader.structure.io Description

Holds the classes needed for reading Protein Data Bank structure files.
